# Influence of radiotherapy dose rate on gold nanoparticle‐induced radiosensitization from high dose‐rate brachytherapy and external beam therapy

**DOI:** 10.1002/mp.70372

**Published:** 2026-02-27

**Authors:** Daniel Cecchi, Nolan Jackson, Sacha Freeman, Kieren O'Neil, Mehran Goharian, Wayne Beckham, Devika B. Chithrani

**Affiliations:** ^1^ Department of Physics and Astronomy University of Victoria Victoria British Columbia Canada; ^2^ British Columbia Cancer Victoria British Columbia Canada; ^3^ Centre for Advanced Materials and Related Technologies (CAMTEC) University of Victoria Victoria British Columbia Canada

**Keywords:** brachytherapy, cancer, gold nanoparticles, radiosensitizer, radiotherapy, therapeutics

## Abstract

**Background:**

Associated normal tissue toxicity from current radiotherapy (RT) treatments limits effective dose escalation in the tumor to achieve nominal treatment results. Gold nanoparticles (GNPs) as radiosensitizing agents to locally increase photoelectron production have gained interest as a safe and viable method to improve therapeutic results. Among many other factors, the dose rate of the incident radiation has been shown to affect the radiosensitizing properties of GNPs significantly.

**Purpose:**

To evaluate GNP‐induced radiosensitization during variable dose rate delivery from the decay of a high dose rate brachytherapy 192‐Ir source and a clinical 6MV linear accelerator (LINAC).

**Methods:**

HEC‐1A endometrial cancer cells were seeded into 35mm petri dishes with or without 10µg/mL with spherical 11nm GNPs functionalized with polyethylene glycol and integrin binding domain RGD to improve intracellular uptake. Variable dose rate delivery from the 192‐Ir source was achieved at two source strengths of 37.95 mGy m^2^/h and 18.97 mGy m^2^/h, corresponding to dose rates of 1.1 and 0.55Gy/min, respectively. For 6MV irradiations, dose rate variability was controlled by adjusting the distance to the target from 91cm to 129cm, yielding identical dose rates of 1.1 and 0.55Gy/min, respectively. Cellular viability was measured using a clonogenic assay after irradiations between 0 and 8Gy, and a DNA double‐strand break assay after 2Gy irradiations.

**Results:**

GNP‐induced radiosensitization was significantly greater with higher dose rates than lower. Clonogenic loss with GNPs was increased from 1.00 to 1.19 (*p <* *0.001*) with higher dose rates from 192‐Ir source and from 1.03 to 1.16 (*p <* *0.001*) with higher dose rate LINAC irradiations. DNA damage increase from GNPs was not significant at lower dose rates for both 192‐Ir (*p *> 0.05) and LINAC (*p *> 0.05) irradiations; however, DNA damage was significantly increased at higher dose rates (192‐Ir: *p *< 0.01; 6MV: *p *< 0.05).

**Conclusions:**

We have successfully demonstrated in vitro that clinically plausible GNP concentrations can induce variable radiosensitization based on the administered dose rate from both 192‐Ir and LINAC irradiations. This work demands future research into the clinical translation of GNPs into high‐dose‐rate environments.

## 1 INTRODUCTION

**FIGURE 1:**
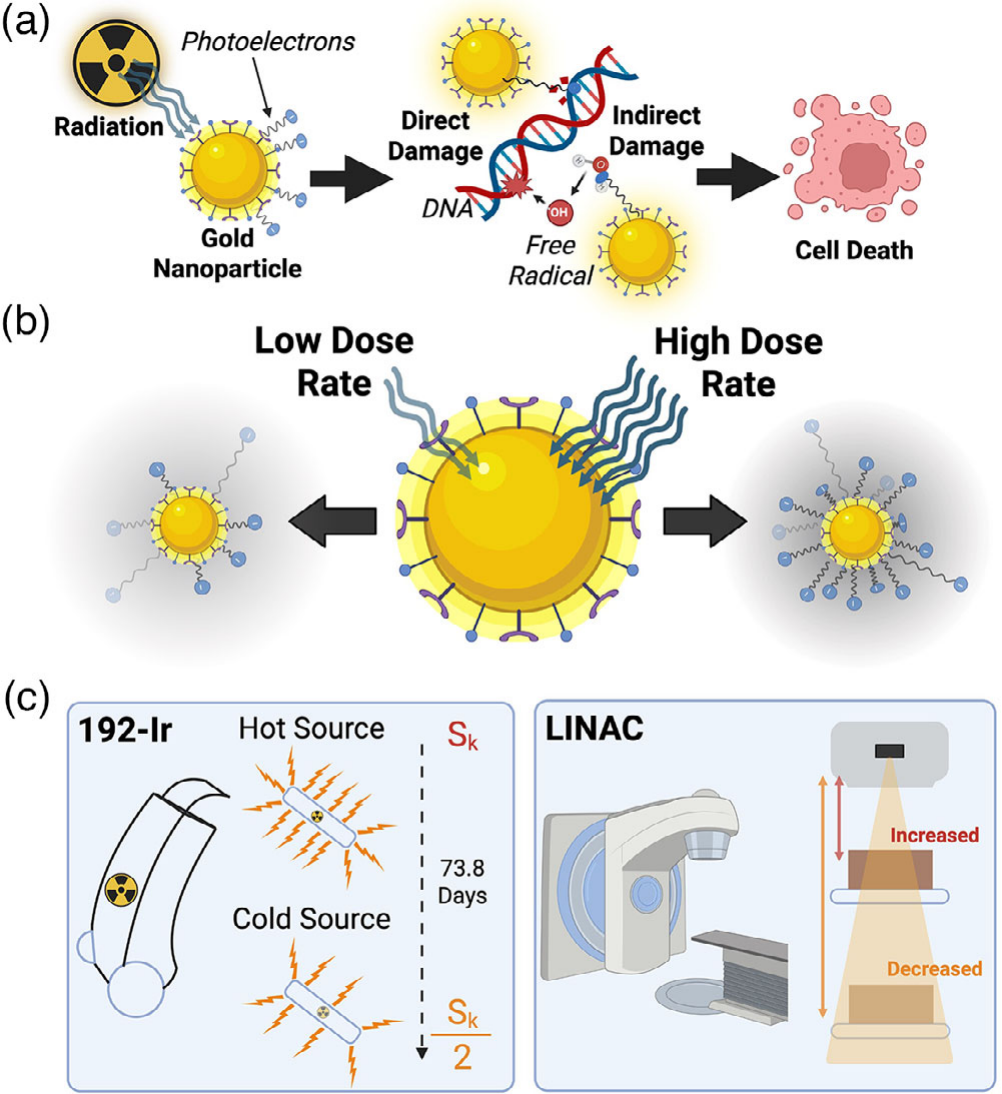
Gold nanoparticle (GNP)‐induced radiosensitization. Direct and indirect DNA damage induction via generated photoelectrons from incident radiation on GNPs leads to loss in cellular viability (a); Variable dose rate delivery incident on GNPs leads to alteration in photoelectron production (b); Brachytherapy source decay and variation in LINAC source‐to‐sample distance leads to a change in averaged dose rate delivery (c). S_k_: air kerma strength.

In recent decades, the advancement of radiation therapy (RT) delivery for cancer treatments via precision targeting and imaging advancements has significantly improved cure rates.^1^, ^2^ Still, healthy tissue exposure is a critical limitation restricting any effective dose escalation in the tumor that could achieve the desired outcomes. Nanoparticles as radiosensitizing agents have gained interest as a possible method to increase cell damage within the tumor. High‐Z gold nanoparticles (GNPs) are some of the most widely researched radiosensitizing agents due to their ease of fabrication, unique surface chemistry for facile functionalization, and biocompatibility.^3^, ^4^, ^5^ Current research has demonstrated their effectiveness in a wide range of clinical scenarios, such as low‐energy brachytherapy and conventional, high‐energy, external beam radiotherapy (EBRT).^6^, ^7^, ^8^ The predominant mechanism expected behind GNP‐induced radiosensitization is the generation of low‐energy, short‐range secondary electrons from the photoelectric effect leading to both direct and indirect DNA damage through molecular bond dissociation and free radical formation, leading to loss in cellular viability (Figure 1a).^4^, ^9^ To this end, low to medium‐energy incident photons are the predominant choice for GNP radiosensitization in the literature, given their increased photoelectric cross‐section relative to soft tissue (∝ Z^3^E^−3^).^10^, ^11^, ^12^, ^13^ Recently, radiosensitization has also been attributed to other factors, such as induced oxidative stress and damage to mitochondrial respiration, demonstrating a multi‐faceted mechanism behind GNP‐induced radiosensitivity requiring further elucidation.^14^, ^15^, ^16^

An increased photoelectric cross‐section produces a greater flux of photoelectrons from incident radiation. These photoelectrons generally have low energy and a short penetration distance into the surrounding tissue, improving local dose deposition. Damage is introduced through their induction of reactive oxygen species from water radiolysis and DNA bond dissociation.^17^ It is, therefore, hypothesized that increasing the rate of photoelectron production could directly increase the generation rate of cell‐damaging species, leading to improved radiosensitization (Figure 1b).^18^, ^19^ On this note, incorporating GNPs into high‐dose rate treatments may prove beneficial. Higher dose rates have indeed been reported in the literature to induce greater radiosensitization in other metal nanoparticles and GNPs.^18^, ^19^, ^20^ Morozov *et al.* ^18^ demonstrated, in vitro, a dose rate dependency on varying GNP sizes irradiated via a 200 kVp orthovoltage beam between 0.5 to 2.0 Gy/min.^18^ The authors showed that smaller, 12 nm bare GNPs showed a proportional increase in radiosensitization with increasing dose rates with high GNP concentrations of 2.4 mg/mL. Similarly, Marques *et al.* ^19^ used thiolated bombesin peptide conjugated 5 nm GNPs dosed at a lower 36 µg/mL to show greater radiosensitization and cell kill with ∼1 Gy/min dose‐rates compared to ∼26 mGy/min obtained from a Co‐60 source.

High‐dose‐rate brachytherapy (HDR) may provide ideal conditions to leverage optimal GNP radiosensitization properties of high dose rates and lower‐energy photon irradiation compared to conventional high‐energy EBRT. HDR‐BT uses high‐activity radioactive sources—typically 192‐Ir with an average emission energy of 397 keV—to target malignant tissue through temporary interstitial or intracavitary implants. Dose rates around 7.5 Gy/min are commonly achieved within 1 cm of the radioactive source, greatly exceeding typical dose rates in conventional EBRT or low‐dose‐rate brachytherapy.^21^, ^22^ Monte Carlo (MC) studies evaluating dose enhancement in simplified systems of varying gold concentrations irradiated via an 192‐Ir source in water show upwards of a 28% increase in dose deposition.^8^, ^23^ However, these results are generally only achievable with a high concentration of gold content (> 5 mg‐Au / g‐H2O) that would not be easily translated clinically due to toxicity concerns. At lower gold concentrations (< 1 mg‐Au/g‐H2O), similar MC studies show little to no dose enhancement.^24^, ^25^ Contrary to these results, many in vitro studies show significant radiosensitization at more clinically plausible concentrations. Yogo et al. showed radiosensitization in plasmid DNA dosed with GNPs at a significantly reduced 64 ng/mL concentration.^7^ More recently it was demonstrated a significant reduction in PC3 and HeLa cancer cell survival after 192‐Ir irradiations from 10 µg/mL GNP concentrations.^26^ This discrepancy between MC and experimental results supports the hypothesis behind a multifaceted mechanism of GNP radiosensitization that is currently not modelled in silico.

To successfully implement GNPs into clinical HDR‐BT workflows, it is necessary to evaluate their relative effectiveness over a radioactive source's lifetime. During this time, the dose rate can drop by nearly 50%, which could significantly affect the reported radiosensitization and treatment efficacy. Yet, no such evaluation has been performed using representative biological systems with clinically plausible gold concentrations. Herein, we present the first in vitro evaluation of a dose rate effect from GNPs during the decay of a common HDR‐BT source over its clinical lifetime—192‐Ir. The successful characterization of the radiosensitization capabilities will help future clinical treatment plans by defining the relative effectiveness of GNPs based on the current strength of the source. Furthermore, we present a similar comparison between conventional EBRT and HDR‐BT found in previous work by reducing the average delivered dose rate from a 6 MV LINAC to match the average dose rate with the 192‐Ir source (Figure 1c).^26^ HEC‐1A cervical cancer cell cultures were employed to assess treatment efficacy as representative tumor cells in HDR‐BT. Cell viability was measured using clonogenic and DNA DSB assays on monolayer cell cultures. By comparing observed cell survival and DNA DSB induction with HDR‐BT compared to conventional EBRT, we offer further insights into the complex mechanism of action for GNP‐induced radiosensitization, closing the gap to their clinical implementation.

## 2 METHODS AND MATERIALS

### 2.1 GNP synthesis, surface modification, and characterization

The GNPs in this study were fabricated following a citrate reduction method.^27^ 1.18 mL of 1% HAuCl_4_ ⋅ H_2_O (Sigma‐ Aldrich, St. Louis, MO, USA) was added to 28.82 mL of dH_2_O and vigorously stirred while brought to a boil. 1.2 mL of 5% sodium citrate tribasic dihydrate (HOC(COONa)(CH2COONa)2·2H2O; Sigma–Aldrich) was then rapidly added to the boiling solution. The liquid quickly changed from clear to dark purple and ruby red, confirming the creation of the GNPs. The solution was left to boil for another 10 min while stirring and then brought to room temperature.

GNPs were PEGylated with PEG of size 2000 Da and an RGD peptide of size 1600 Da. PEG was stirred into the GNP solution so that the grafting density would be 1 PEG molecule per nm^2^ surface area. The peptide containing integrin binding domain RGD was then added to the solution at a density to achieve a ratio of 2 PEG molecules per 1 RGD molecule. The GNP‐PEG‐RGD complex will be denoted simply as GNP in the remainder of the paper. Bare and functionalized GNPs were characterized using ultraviolet‐visible spectrometry (λ Spectrophotometer, Perkin Elmer, Waltham, MA, USA) for size and concentration measurements. In addition, dynamic light scattering (DLS) and ζ‐potential (LiteSizer 500, Anton Paar, Graz, Austria) were used to verify GNP functionalization and surface charge. DLS, ζ, and UV‐VIS measurements were performed in triplicate to determine statistical error of the measurement and polydispersity index (σ/√N). Transmission electron microscopy (TEM) images were taken to verify the diameter of the GNPs.

### 2.2 2‐Dimensional cell culture growth and intracellular GNP uptake

Endometrial cancer cell line, HEC‐1A (ATCC#: HTB‐112), was purchased from the American Type Culture Centre (ATCC). Cells were cultured in T‐75 flasks using high glucose Roswell Park Memorial Institute 1640 Medium (RPMI; Gibco) supplemented with 2 mM GlutaMax (Gibco), 10% Fetal Bovine Serum (Gibco) and 1% penicillin/streptomycin (Gibco). Cell cultures were washed using phosphate‐buffered saline (PBS), and TrypLE (Gibco) was used for cell detachment. Cells were incubated at 37°C with 5% CO_2_ and subcultured every 3–5 days once ∼80‐90% confluency was achieved.

Cells were initially split from a monolayer at approximately 80% confluency. Cells were plated to achieve a final confluency of approximately 70% in 35 mm Petri dishes. Once plated, cells are left in the incubator at 37°C and 5% CO_2_ for 24 h to ensure adherence, after which experiments are initiated.

An inductively coupled plasma mass spectrometry (ICP‐MS) technique was used to quantify intracellular GNP uptake. Cell cultures were dosed with PEG and RGD functionalized GNPs at a concentration of 10 µg/mL and left to incubate at 37°C and 5% CO_2_ for 24 h following treatment. Cells were then rinsed three times with PBS and trypsinized for counting. To measure the gold content for each sample, 500 µL of each condition were treated with 250 µL aqua regia (3:1 ratio of HCl:HNO3) in a 90°C mineral oil bath for a minimum of 60 min. After, 100 µL of hydrogen peroxide was added to the samples, which were left in the oil bath for an additional 30 min to ensure complete cell breakdown. Deionized water was added to dilute the acid content to 2.5% v/v. The absolute gold content in each sample was quantified using ICP‐MS (8800 Triple Quadrupole, Agilent, Santa Clara, CA, USA). Calculations of intracellular nanoparticle content based on measured absolute gold content can be found in Supplemental Information.

GNP uptake in the cellular membrane was also qualitatively verified using live‐cell and hyperspectral imaging (HSI). Citrate‐capped GNPs were functionalized with Cy5‐labeled PEG and RGD at a 2:1 ratio before dosing procedures. 2‐D cell cultures were created as described previously and dosed at 10 µg/mL with the fluorescently labeled GNP‐complex for 24 h NucBlue™ Live ReadyProbes™ Reagent (R37605; ThermoFisher Scientific, Waltham, MA, USA) containing Hoechst 33 342 dye was used to stain nuclei before imaging. Images were taken using a 60 x oil immersion objective lens using a confocal laser scanning microscope (Zeiss LSM 980, Carl ZeissMicroscopy GmbH, Jena, Germany). Hyperspectral imaging (HSI) and darkfield imaging were performed to characterize GNP uptake in cellular membranes further.

### 2.3 Dosimetry

HDR‐BT treatment delivery setup can be seen in Figure S1. HDR irradiations were performed with an 192‐Ir Flexisource and an Elekta Flexitron HDR Afterloader (Model: 136149, Elekta, VEENENDAAL, Netherlands). For in vitro HDR irradiations, a modular Solid Water phantom was created and validated in previous work to deliver a uniform dose across the face of a 35 mm petri dish from a 200 cGy prescription.^26^ This research performs a similar analysis by irradiating Gafchromic EBT‐4 radiochromic film (lot #: 12182303) at 500, and 800 cGy dose prescriptions. Post‐irradiation, the film was left to saturate for 24 h, after which it was read in the red channel using an Epson Expression 10000XL (Epson, Suwa, Japan) and processed on FilmQA Pro v.7 (Ashland Inc., Wayne, New Jersey, USA). Absolute dosimetry was accomplished by applying a calibration curve generated from the 192‐Ir source as described previously.^26^ Radiochromic film dosimetry is shown in Figure S2, confirming uniform dose delivery at higher doses.

To achieve variable dose rates from the 192‐Ir source, samples were irradiated with either a hot or cold source corresponding to a source air kerma strength (S_k_) of 37.95 mGy m^2^/h and 18.97 mGy m^2^/h, respectively. The average delivered dose rate was reported simply as the total delivered dose according to the treatment plan divided by the reported treatment delivery time on the treatment unit. High dose rate and low dose rate irradiations corresponded to 1.1 and 0.55 Gy/min, respectively, and are labeled accordingly as *h*‐HDR‐BT and *l*‐HDR‐BT.

LINAC irradiations were accomplished with a 6 MV medical LINAC (Varian Truebeam, Palo Alto, CA, USA) after fabricating a Solid Water expansion for the HDR phantom to incorporate greater side scatter, backscatter, and buildup (Figure S3). The apparatus was CT‐scanned on a GE Healthcare CT Scanner (GE Healthcare, California, USA) and imported into Eclipse TPS 15.606 (Varian Medical Systems, Palo Alto, California, USA), where a 200 cGy dose prescription was set to the base of the petri dish (target volume). 220 MU was determined to deliver 200 cGy dose to the sample at a 5 cm depth, 10 × 10cm field size, and 95 cm source‐to‐surface distance. For larger doses, the delivered MU was scaled proportionally. To match the average delivered dose rates to the base of the petri dish of 1.1 and 0.55 Gy/min from the 192‐Ir source, MU hand calculations were conducted to determine the corresponding source‐to‐sample distance (SD_sample_) required for each dose rate with a constant depth of 5 cm and LINAC output of 100 MU/min. Tissue Maximum Ratio (TMR)‐based MU hand calculations to determine the required SD_sample_ are shown in Supplemental Information along with the total delivered MUs for each dose and dose rate. High and low dose rate LINAC irradiations are labeled *h*‐LINAC and *l*‐LINAC, respectively.

### 2.4 In vitro cellular irradiations

2D cell culture preparation was performed as described in Section 2.2. 24 h before irradiation, cell cultures were dosed with functionalized GNPs at 10 µg/mL. After irradiation, the media was removed, the cell cultures were washed twice with 1 mL of PBS, and 3 mL of fresh media was added back to each well. The samples were immediately prepped for analysis and post‐processing or left in the incubator for a predetermined time. Samples that are not treated with GNPs are labeled as control (CTRL).

#### 2.4.1 Clonogenic assay

Cells were seeded in a 35 mm petri dish and incubated for 24 h, followed by the dosing procedure described previously. After irradiation, samples were washed three times with PBS and trypsinized for cell counting. Fresh media was added to the Petri dishes to dilute the trypsin, and the cell suspension was transferred to an autoclaved 1.5 mL Eppendorf tube. After cell counting, the required number of cells was added to a 60 mm Petri dish in triplicate for colony formation. HEC‐1A cells were plated at 100, 300, 500, 1000, 4000, 5000, 8000, and 10 000 per plate for 0, 100, 200, 300, 400, 500, 600, and 800 cGy, respectively. Plating efficiency (0 cGy) was determined for each dose rate and irradiation modality. LINAC irradiations, used the same unirradiated sample as control for both *h‐*LINAC and *l‐*LINAC irradiations because they were completed on the same day and cells were cultured from the same passage, which was not possible with HDR‐BT irradiations. Post‐irradiation, the seeded cell cultures were left to incubate for 15 days for colony growth. After the incubation period, the media was poured out, and the cells were stained with 0.5% methylene blue for 20 min, after which the stain was washed with dH_2_O, and the plates were allowed to air‐dry overnight. Using a 10X microscope, colonies were counted for plating efficiency (PE) and survival fraction (SF) statistics. A colony formed from a single viable cell was defined as a cluster of at least 50 cells.^28^ PE was calculated by dividing the total number of colonies formed without radiation exposure by the number of cells seeded; the SF was calculated by dividing the total number of colonies formed after irradiations by the number of cells seeded and dividing by the PE of the respective condition without irradiation (0 cGy). The cell survival as a function of dose was then fitted to the linear‐quadratic (LQ) model (further described in Section 2.6). GNP‐induced radiosensitization was evaluated using the sensitization enhancement ratio (SER) defined below:

(1) $$SER=\frac{Dose\;for\;10\%\;SF\;w/o\;GNPs}{Dose\;for\;10\%\;SF\;w/\;GNPs}$$

whereby the 10% SF dose for each condition was determined using the fitted LQ model.

#### 2.4.2 Radiation‐Induced DNA damage assay

Cells were grown on glass coverslips in 35 mm petri dishes at densities described in Section 2.4 and were incubated for 24 h for adherence. GNP‐dosing procedures also followed, as described in Section 2.4. Post‐irradiation to 200 cGy, the media in each well was removed and replaced with fresh media before cells were incubated. 24 h after irradiation, cells were fixed with 4% PFA for 5 min at room temperature, followed by two PBS washes for 5 min each. Cells were blocked with 2%BSA/0.1% Triton‐X in PBS for 20 min. The primary antibody 53BP1 was diluted to 1:200 in 0.5%BSA/0.1%Triton‐X/PBS, while the second antibody (Alexa Fluor 488; excitation 490 nm, emission 525 nm) was diluted to 1:500 in 0.5% BSA/0.1% Triton‐X/PBS. The coverslips were placed face down onto 50 uL of the primary antibody on parafilm and incubated in the dark for 60 min. The coverslips were returned to the Petri dishes and washed with PBS for five min and once with 0.5% BSA/0.1% Triton‐X/PBS. On new parafilm, the coverslips were placed face down in 50 uL of the secondary antibody solution and incubated in the dark for 45 min. Coverslips were returned to the wells, washed with PBS, and mounted to glass microscope slides with ProLong™ (P36930; ThermoFisherScientific, Waltham, MA, USA) Glass Antifade Mountant for imaging. 53BP1 foci were imaged using a 60X oil immersion lens and a confocal laser scanning microscope (Zeiss LSM 980). Images were processed, and foci and nuclei were counted. GNP‐induced DNA damage‐enhancement was quantified via the relative increase in average foci‐per‐cell, representing sites of DNA DSB repair.

### 2.5 Temporal variation in dose rate delivery

During HDR‐BT and conventional EBRT, the delivered dose rate has significant variability with the distance of the sample to the X‐ray source. This could result in variable radiosensitization across the target volume, leading to the question of whether the average or maximum dose rate achieved during treatment has a more significant effect on radiosensitization. To determine the instantaneous dose rate across the sample during 192‐Ir irradiations, Monte Carlo (MC) simulations were conducted using TOol for PArticle Simulation (TOPAS) v3.9, which has previously been validated for use with low‐energy, high‐activity radioactive sources.^29^ The Solid Water phantom was simulated as a 10 × 10 × 4 cm block of water with the base of the petri dish as a flat film of water, 1 mm in depth, centered in the X and Y dimensions of the phantom, with its base positioned 1.5 cm from the top. Voxel size was set to 35 mm/ 50 Bins in both the X and Y directions. Each dwell position and time was simulated according to the treatment plan generated by the treatment planning system Oncentra™. The number of particle histories at each position was changed according to the dwell time and the S_k_ of the source at the time of treatment plan creation using the following formula, where Γ = 4.037 cGy cm^2^/h/mCi was used based on the manufacturers specifications:

(2) $$\begin{array}{cc} Num.\;Particle\;Histories & =T_{Dwell\;}\;\left[s\right]\cdot S_{k}\left[mGy\frac{m^{2}}{hr}\right]\\ & \cdot \Gamma^{-1}\cdot 3.7\times 10^{10}\left[\frac{particles/s}{Ci}\right] \end{array}$$

Dose rate delivery was calculated by dividing the total dose delivered to a point at the base of the petri dish by the source's dwell time during the irradiation:

(3) $$Dose-Rate\;\;\left[\frac{Gy}{s}\right]=\frac{Dose\;delivered\;to\;voxel\;\left[Gy\right]}{T_{Dwell}\left[s\right]}\;$$

To determine the instantaneous dose rate achieved during LINAC irradiations, we estimated the pulse rate and dose per pulse based on tabulated measurements from Varian TrueBeam documentation as a 4 µs pulse width and 0.03 MU/pulse. The dose‐per‐pulse can then be estimated using the known delivered dose and total MUs.

### 2.6 Statistics

In vitro experiments were conducted in triplicate to attain statistical significance estimates. Error bars on individual sample measurements are defined as ± 1σ. Clonogenic survival data were fitted to the LQ model describing cell survival fraction as a function of dose (D):

(4) $$SF=e^{-D\left(\alpha -\beta D\right)}\;$$

The α and β parameters were estimated for each group after the data was fitted using a non‐linear least‐squares method that employed a Levenberg–Marquardt (LM) algorithm (Scipy.optimize, v1.16.1). A parametric bootstrapping procedure was performed to calculate the 95% confidence interval (CI) of the parameters, along with the SER (defined in Equation 1). 1000 iterations created a synthetic dataset, from which a distribution of *α*, *β*, *α/β*, and SER values was created. The LQ parameters and SER will be reported from the measured dataset, and not the mean of the synthetic, bootstrapped dataset; however, the 95% CI from the bootstrapped distribution will be given.

The significance of the SER value will be concluded if SER = 1 is not contained in the 95% CI generated from the bootstrapped distribution. Similarly, to compare radiosensitization between individual groups, the difference in SER (*Δ*) was computed for each bootstrap iteration between paired groups. A 95% CI was obtained as described previously, and statistical significance was concluded if *Δ* = 0 was not contained in the interval. A two‐sided p‐value was then calculated as twice the fraction of differences that are greater or less than SER = 1 or *Δ* = 0. Statistical significance was defined as *α* = 0.05.

The effect of GNPs on DNA damage was achieved using a two‐tailed Student's *t*‐test with equal variance on the measured foci/cell averages per condition. Statistical significance was defined as *α* = 0.05.

## 3 RESULTS

### 3.1 GNP‐complex characterization

**FIGURE 2:**
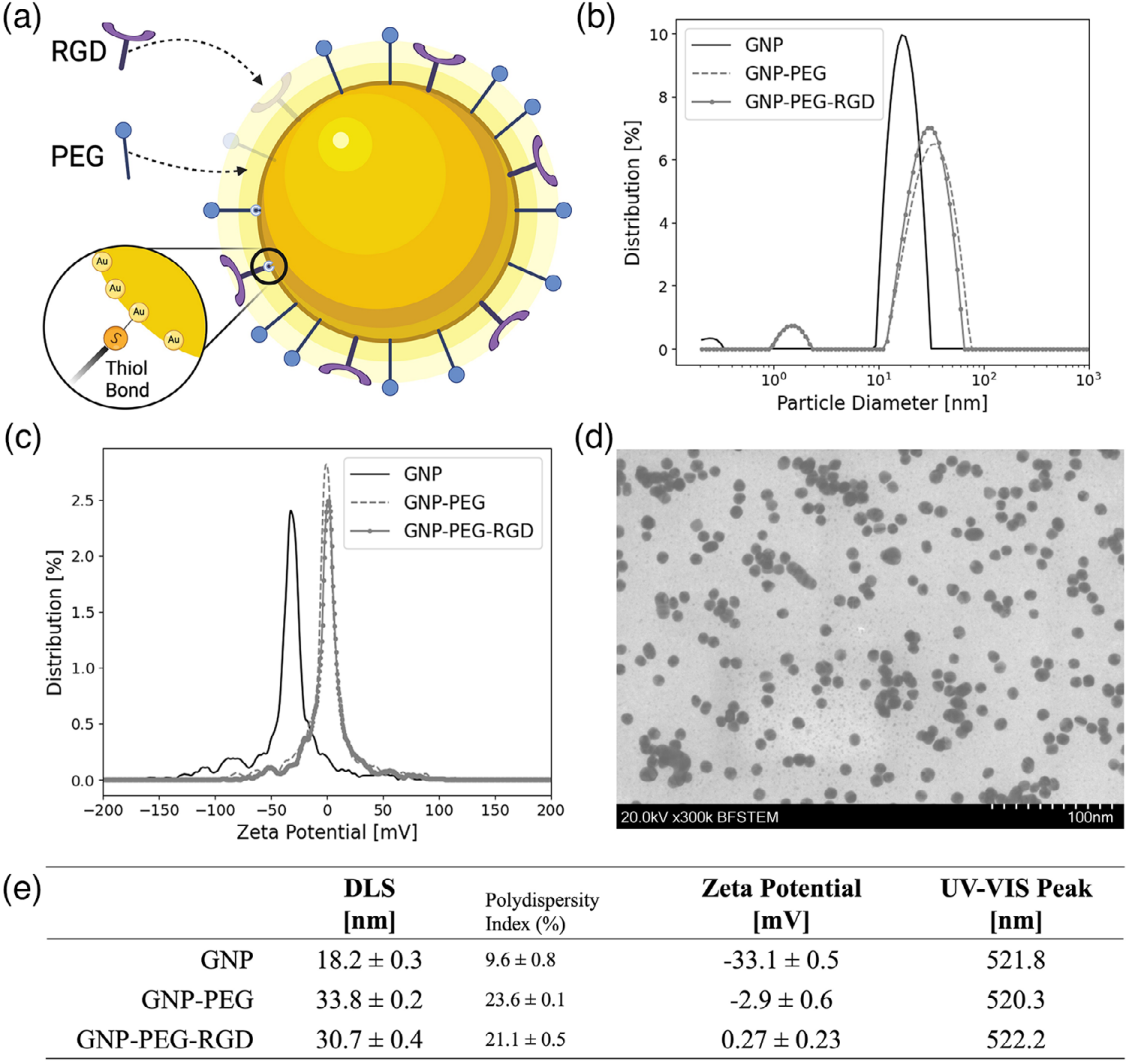
GNP Characterization and functionalization. (a) GNP model with PEG and RGD functionalization. (b–c) DLS and zeta potential measurements of bare and functionalized GNPs. (d) Transmission electron microscopy (TEM) image of functionalized GNPs. (e) Raw DLS, zeta, and UV‐VIS measurements of bare and functionalized GNPs.

DLS, Zeta, and UV‐VIS measurements were performed at each stage of the functionalization process (Figure 2a) to qualitatively verify GNP fabrication and functionalization. As shown in Figure 2b, the functionalization of the GNPs with both PEG and RGD increased the nanoparticle's hydrodynamic diameter from 18.2 nm ± 0.3 nm to 30.7 nm ± 0.4 nm, with a polydispersity index of 9.6% ± 0.8% and 21.1% ± 0.5%, respectively, in accordance with previously published research.^30^, ^31^ It would be expected that the functionalization with RGD after PEGylation would not lead to a significant difference in the measured hydrodynamic diameter, as we have reported here (Figure 2e). Although this difference is small and could be a result of experimental variability, it could also suggest subtle conformational changes in PEG due to the addition of RGD to the surface; however, this was not verified nor expected to significantly affect our results. Small (∼1 nm), unaggregated gold clusters are observed in GNP and GNP‐PEG‐RGD solutions (Figure 2b) though are not expected to significantly affect our results due to their low abundance (< 1 %) compared to larger spherical GNPs. In addition, zeta potential measurements were performed on the GNP colloidal solution (Figure 2c). Citrate‐capped GNPs exhibit strong negatively charged surface potential due to the citrate molecules. Upon functionalization with neutral PEG and RGD, the zeta potential is shifted from −33.1 mV for bare GNPs to 0.27 mV with functionalized GNPs. UV‐VIS measures the absorbance of a sample for a given incident light relative to a blank sample. For citrate‐capped GNPs, the spectral absorbance peaked at 521.8 nm, which was red‐shifted to 522.2 nm with the functionalization with PEG and RGD. TEM images of citrate‐capped GNPs confirm the core diameter at 13.6 nm ± 1.21 nm (Figure 2d). The diameter of the GNPs was also confirmed using UV‐VIS measurements, as proposed by Haiss *et al*.^32^ The ratio of the absorbance of the GNP solution at the wavelength (*λ*) of peak absorbance and at *λ* = 450 nm was found to directly relate to the mean diameter of the GNPs in the solution. Based on the absorbance spectra of the colloidal solution (Figure S4), the mean diameter of the bare, unfunctionalized GNPs is reported as 11 nm, with an estimated error—according to Haiss *et al.—*of 18% (2.0 nm). After functionalization of a GNP of size 11 nm, the surface was coated with approximately 400 PEG molecules to 200 RGD molecules per GNP; however, the specific packing density of the ligands was not directly verified.

### 3.2 Intracellular GNP uptake in 2‐dimensional tissue cultures

**FIGURE 3:**
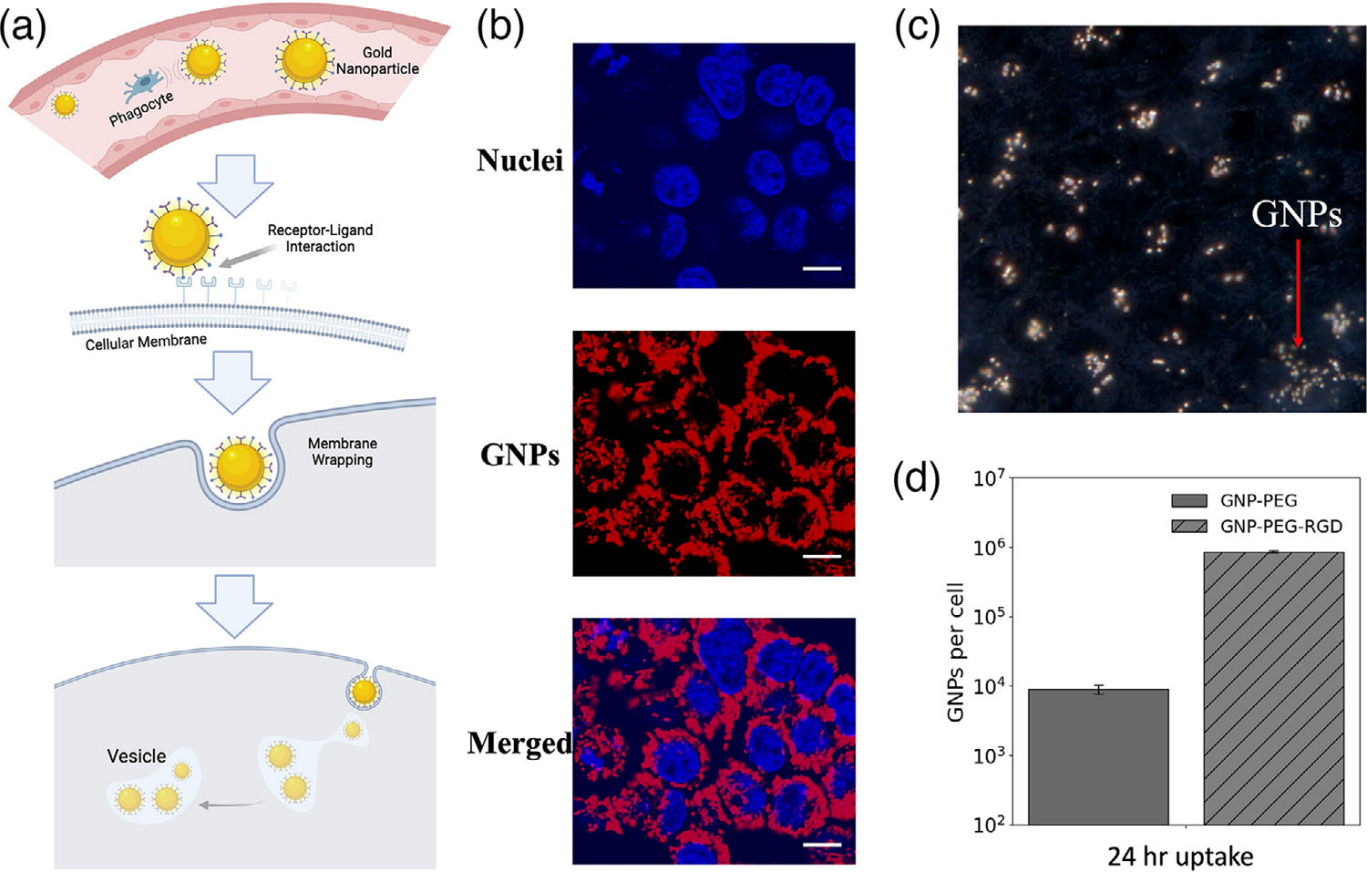
Receptor‐mediated endocytosis of gold nanoparticles (GNP). Endocytotic pathway of GNPs into the intercellular matrix via membrane wrapping (a); Live‐cell images of Dapi stained nuclei in blue and GNPs in red of HEC‐1A cells (b); Darkfield, HSI images highlighting GNPs within the cellular membrane (c); Gold content quantification in HEC‐1A cells dosed with 10 µg/mL GNP complex from ICP‐MS (d).

GNP uptake within the cellular membrane is facilitated by receptor‐mediated endocytosis (Figure 3a).^33^ PEGylation of the GNPs enables improved blood circulation time in vivo but limits nanoparticle uptake.^34^ Integrin binding domain RGD was grafted to the surface of the GNPs to target the αvβ3 integrin overexpressed by many cancer cells.^3^, ^35^, ^36^ Live‐cell imaging and darkfield HSI images were acquired to verify that the nanoparticles were being endocytosed rather than accumulating on the membrane surface (Figure 3b,c). Citrate‐capped GNPs were PEGylated with Cy5‐labeled PEG along with the RGD peptide at the same 2:1 ratio to verify intracellular GNP uptake qualitatively. Figure 3b shows live‐cell images of samples dosed 24 h prior with the fluorescently labeled GNPs, confirming GNP presence within the cellular membrane and surrounding the nucleus. HSI images of fixed cells were also acquired under a coverslip under identical GNP dosing conditions (Figure 3c). Quantitative verification of GNP‐uptake was verified using ICP‐MS (Figure 3d), which reported an average of 8.5 × 10^5^ and 9 × 10^3^ GNPs/cell when functionalized with and without RGD, respectively, demonstrating significantly greater intracellular uptake with the targeting motif.

The 10 µg/mL dosing concentration was chosen to limit toxicity in future in vivo experiments. The dosing concentration approximately translates to 1–2 mg/kg in vivo for a 20 g mouse with ∼1–2 mL of blood, which is well below the LD50 of GNPs reported for mice.^37^ Monolayer cell cultures are limited in their biological relevance but the dosing concentration can be translated to more complex biological systems like 3‐dimensional spheroids and in vivo with limited toxicity.^6^, ^30^

### 3.3 In vitro irradiations

**FIGURE 4:**
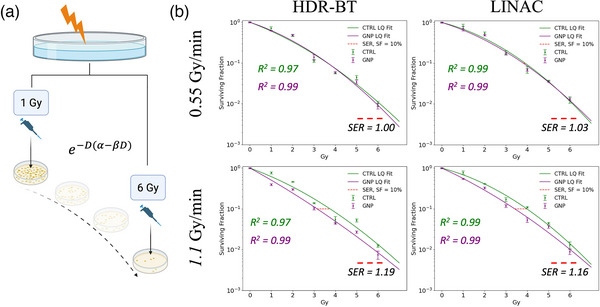
Clonogenic survival after high and low dose rate irradiation. Clonogenic plating procedure after 0–6 Gy irradiations from either HDR‐BT or LINAC (a). Cell survival curves with and without GNPs at 0.55 and 1.1 Gy/min for HDR‐BT (left) and LINAC (right) irradiations (b).

Clonogenic survival post‐HDR‐BT and LINAC irradiations is shown in Figure 4, with individual colonies depicted in Figure S5. After HDR‐BT, GNPs induced an SER of 1.19 [95% CI 1.13–1.25] (*p* < 0.001) and 1.00 [95% CI 0.96–1.05] (*p* = 1.0) from the 1.1 Gy/min and 0.55 Gy/min dose rates, respectively, resulting in an approximate 19% (*p < *0.001) increase in GNP‐induced radiosensitization with higher dose rates during HDR‐BT irradiations. After *h*‐LINAC *and l*‐LINAC irradiations, GNPs induced an SER of 1.16 [95% CI 1.12–1.22] (*p* < 0.001) and 1.03 [95% CI 0.97–1.07] (*p* = 0.43) from the 1.1 Gy/min and 0.55 Gy/min dose rates, respectively, indicating an approximate 13% (*p < *0.001) increase in GNP‐induced radiosensitization with higher dose rates from high‐energy conventional EBRT. These findings are similar to reported SERs in the literature, despite using significantly lower gold concentrations.^18^, ^38^, ^39^ From lower dose rate treatments for both treatments, the SER was not statistically significant, indicating that the administration of GNPs during HDR‐BT treatments near the end of the source's clinical lifetime may not be efficacious; however, this result needs to be further tested in vivo and clinical trials.

Fitting the LQ model to clonogenic survival data can help provide further insights into the induction of damage caused by radiation or other interventions.^40^, ^41^ The α parameter [Gy^−1^] is often interpreted as damage caused by lethal, single‐track events, which leads to irreparable DNA damage such as DNA DSBs that occurs linearly with dose. Meanwhile, the β parameter [Gy^−2^] is interpreted as sublethal damage that requires the interaction of two single‐track events and is thus quadratic in dose. The ratio of the two, termed the α/β ratio [Gy], defines the radiosensitivity of the tissue, whereby a larger ratio indicates greater radiosensitivity than a comparably lower ratio. Presented in Figure 4b are samples irradiated up to 6 Gy due to the relevance of the LQ‐model at these dose prescriptions.^42^ An 8 Gy dose delivery was also performed and the LQ‐fit between 0–8 Gy is included in Figure S6a,b along with the measured α, β, and SER parameters. The multi‐target‐single hit (MTSH) and Two‐Component (TC) models were also evaluated for goodness‐of‐fit on the clonogenic data between 0–8 Gy and are included in Figure S7.

The α and β parameters from the LQ‐fit in Figure 4 are listed in Table 1. No significant differences are reported for both parameters between the CTRL and GNP samples at low dose rates for both HDR‐BT and LINAC (*p *> 0.05). At lower dose rates, the α parameter is significantly larger after *l‐*HDR‐BT irradiations without GNPs compared to *l‐*LINAC irradiations (*p *= 0.01)—indicating potentially greater single‐track events from lower energy radiation; however, the α value was not significantly different for GNP‐treated samples between *l*‐HDR‐BT and *l*‐LINAC irradiations (*p* = 0.6). Meanwhile, the β parameter is similar in both GNP‐treated and untreated samples and between irradiation energies (*p > *0.8). No significant differences were found in the α/β ratio between CTRL and GNP samples post‐low dose rate irradiations for both HDR‐BT and LINAC treatments (*p *> 0.3). Similarly, no significant difference is found in this parameter when comparing *l‐*HDR‐BT and *l*‐LINAC irradiations (*p* = 0.1). This result shows minimal effect of GNPs on both HDR‐BT and LINAC irradiations at the dose rate of 0.55 Gy/min on HEC‐1A cells.

**TABLE 1:** α and β values from LQ‐model fitted to clonogenic survival data. 95% confidence interval (CI) measured using bootstrapped procedures provided below each value.

0.55 Gy/min	HDR‐BT		LINAC	
	CTRL	GNP	CTRL	GNP
α 95% CI	0.45 (*0.41*, *0.49*)	0.40 (*0.35*, *0.46*)	0.31 (*0.24, 0.37*)	0.37 (*0.34*, *0.41*)
β 95% CI	0.05 (*0.04, 0.06*)	0.06 (*0.05*, *0.08*)	0.07 (0.06, 0.09)	0.06 (*0.05*, *0.07*)
α / β 95% CI	9.3 (*6.8*, *12.1*)	6.4 (*4.6*, *8.8*)	4.3 (*2.8*, *6.3*)	6.3 (*5.1*, *7.6*)

**1.1** **Gy/min**	HDR‐BT		LINAC	
	CTRL	GNP	CTRL	GNP
α 95% CI	0.44 (*0.40*, *0.48*)	0.66 (*0.61, 0.71*)	0.32 (*0.26*, *0.36*)	0.55 (*0.51*, *0.60*)
β 95% CI	0.05 (*0.04*, *0.05*)	0.02 (*0.01*, *0.04*)	0.07 (*0.05*, *0.08*)	0.04 (*0.03*, *0.05*)
α / β 95% CI	9.5 (7.5, 11.5)	37.1 (*16.3, 67.2*)	4.9 (*3.3, 6.6*)	16.2 (*11.2*, *23.3*)

During higher dose rate irradiations at 1.1 Gy/min, more deviation between the HDR‐BT and LINAC irradiations is found in both the α and β parameters. Under CTRL irradiations (without GNPs), both the α and β parameters were statistically similar post 1.1 and 0.55 Gy/min dose rates. This result indicates no dose‐rate effect from the delivery type is observed without GNPs, as should be expected.^43^, ^44^ Indeed, Figure S8 in the supplemental information depicts cell survival post HDR‐BT and LINAC irradiations at both dose rates, showing insignificant differences between both curves. The introduction of GNPs led to a significant increase in the α parameter for both *h‐*HDR‐BT (*p* < 0.001) and *h‐*LINAC (*p* < 0.001) irradiations. In contrast, the β parameter significantly decreased compared to CTRL samples for *h*‐LINAC irradiations (*p *= 0.02) but was not significantly different for *h*‐HDR‐BT (*p *= 0.13). This result could be related to the photon energy spectrum and a greater probability of interaction leading to sub‐lethal damage with HDR‐BT irradiations compared to EBRT; therefore, with the induction of GNPs increasing the α parameter and single‐track events, the β parameter may not be affected to the extent that has been measured with LINAC irradiations. The larger α and α/β values with HDR‐BT compared to EBRT is likely due to the lower photon energy spectrum from the 192‐Ir source. While both are conventionally considered low‐LET radiation, the relative increase short‐range secondary electrons at lower photon energies may increase the importance of the α component, leading to higher apparent α/β values as we have reported here.

The α/β parameter was similarly affected with the introduction of GNPs. No statistically significant differences in the α/β ratio were observed with GNPs across the two dose rates examined for either HDR‐BT or LINAC irradiations (*p* > 0.05). However, higher dose rates coupled with GNPs significantly increased the α/β ratio compared to lower dose rates for both HDR‐BT (*p* = 0.01) and LINAC (*p* < 0.01) irradiations. Notably, while no difference in the α/β ratio was found between CTRL and GNP treated samples at 0.55 Gy/min, a significant increase of 290% (*p* < 0.001) and 230% (*p *< 0.001) was observed for HDR‐BT and LINAC irradiations, respectively, at a dose rate of 1.1 Gy/min. As expected, the largest α/β ratio of 37.1 occurred under *h*‐HDR‐BT irradiations. However, there was no significant differences in the α/β ratio between *h‐*HDR‐BT and *h*‐LINAC irradiations (*p *= 0.32) similar to the reported SER from the measured clonogenic data. Proportionally larger α, and α/β parameters in GNP‐treated samples are reported with *h*‐HDR‐BT irradiations compared to *h‐*LINAC, which we suggest is attributable to the varied photon energy spectrum of the two radiation sources. 192‐Ir, with its average emission energy around 400 keV is closer to the k‐edge of Au (∼80.7 keV) compared to a clinical 6 MV photon beam (average energy ∼1.2 MeV). This would lead to a proportionally greater photoelectric cross‐section with 192‐Ir irradiations and thus a larger α and α/β value. MC studies indicate that the GNP concentrations used are insufficient to produce substantial physical dose enhancement; therefore, the radiosensitization observed in this study is likely driven by radiobiological mechanisms that amplify the differential response between 192‐Ir and 6 MV LINAC irradiation.

#### 3.3.1 DNA DSB assay

**FIGURE 5:**
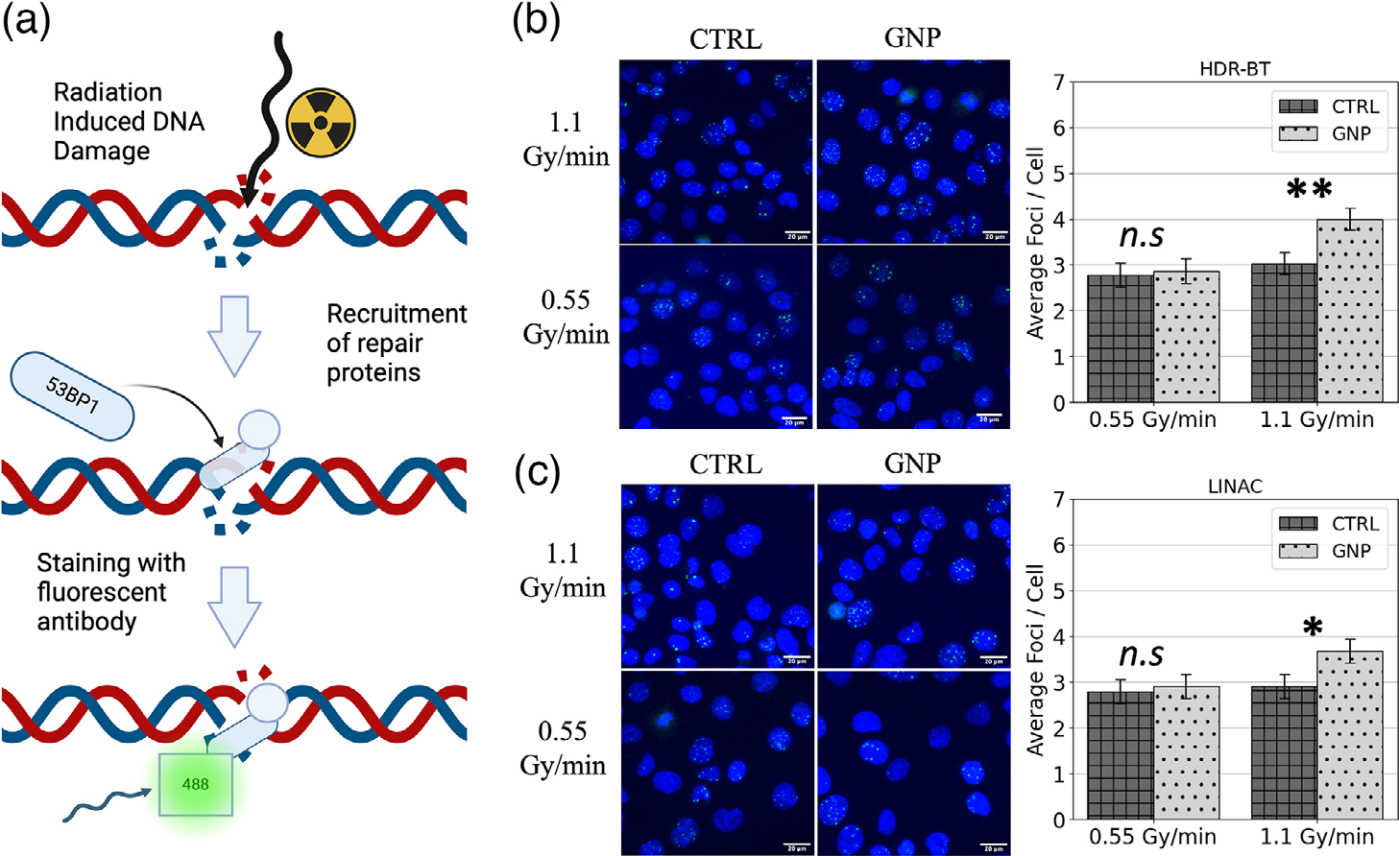
Radiation‐induced DNA damage. DNA damage, localization and antibody staining (a). Confocal images of dapi‐stained nuclei (blue) and DNA damage (green) along with averaged foci counts per cell for 192‐Ir (b) and 6MV (c) irradiations. n.s.: not significant; *: p < 0.05; **: p < 0.01.

An immunohistochemistry assay was used to probe radiation‐induced DNA DSBs, furthering our evaluation of GNP‐induced radiosensitization (Figure 5a). Upon DSB induction, a signaling cascade begins to initiate the recruitment of DNA repair proteins. Among them, a recruited protein, 53BP1, localizes around sites of DNA DSBs and signals non‐homologous end‐joining—an error‐prone DSB repair pathway. Damage fixation occurred 24 h post‐irradiation to measure residual DSBs, which often go unrepaired. The 53BP1 protein is then labeled with fluorescent antibodies to quantify the number of DSBs per cell. Shown in Figure 5b,c are confocal images of DAPI‐stained nuclei in blue and sites of DSB repair in green for high and low dose rates from both HDR‐BT (b) and LINAC (c) irradiations. Shown in Figure S9 are confocal images of un‐irradiated cells, depicting no additional DSB induction from the administration of the GNPs.

DNA DSBs significantly increased with higher dose rates for both HDR‐BT and LINAC irradiations with GNPs compared to untreated samples (Figure 5). From GNP + *h‐*HDR‐BT irradiations, DSBs increased by 32% (Avg. foci/cell GNP: 4.0; CTRL: 3.0; *p < 0.05*) as opposed to only 4% (Avg. foci/cell GNP: 2.9; CTRL: 2.8; *p > 0.05*) from GNP + *l‐*HDR‐BT irradiations (Figure 5b). For GNP *+ h*‐LINAC irradiations, DSBs increased by 26% (Avg. foci/cell GNP: 3.7; CTRL: 2.9; *p < 0.05*) as opposed to only 4% (Avg. foci/cell GNP: 2.9; CTRL: 2.8; *p > 0.05*) from GNP + *l‐*LINAC irradiations (Figure 5c).

### 3.4 Temporal variation in dose rate delivery

During both high and low‐energy irradiation conditions in this study, as is done in the current literature, only the average dose rate across the face of the petri dish has so far been reported. However, both irradiation methods have significant variability in their administered dose rate during treatment delivery, which could theoretically affect radiosensitization capabilities of GNPs. For instance, during 192‐Ir irradiations, the dose rate depends not only on the current S_k_ of the source but also on the proximity of the GNPs to the source, which continually changes during treatment delivery depending on the dwell position. Similarly, LINAC delivery is not continuous but rather in sequential pulses governed by the internal pulse rate of the LINAC. During each pulse, the instantaneous dose rate could significantly exceed the average dose rate during treatment delivery. From the LINAC dose rate of 100 MU/min, estimated MU‐per‐pulse of 0.08 MU/pulse, total delivered MUs of 182.3 MU/200 cGy, the estimated dose‐per‐pulse resulted as 0.072 cGy/pulse. Each pulse is approximately 4 µs long, resulting in a final instantaneous dose rate of approximately 180 Gy/s, or 1.1 × 10^3^ Gy/min for *h‐*LINAC irradiations, or 550 Gy/min for *l*‐LINAC irradiations.

**FIGURE 6:**
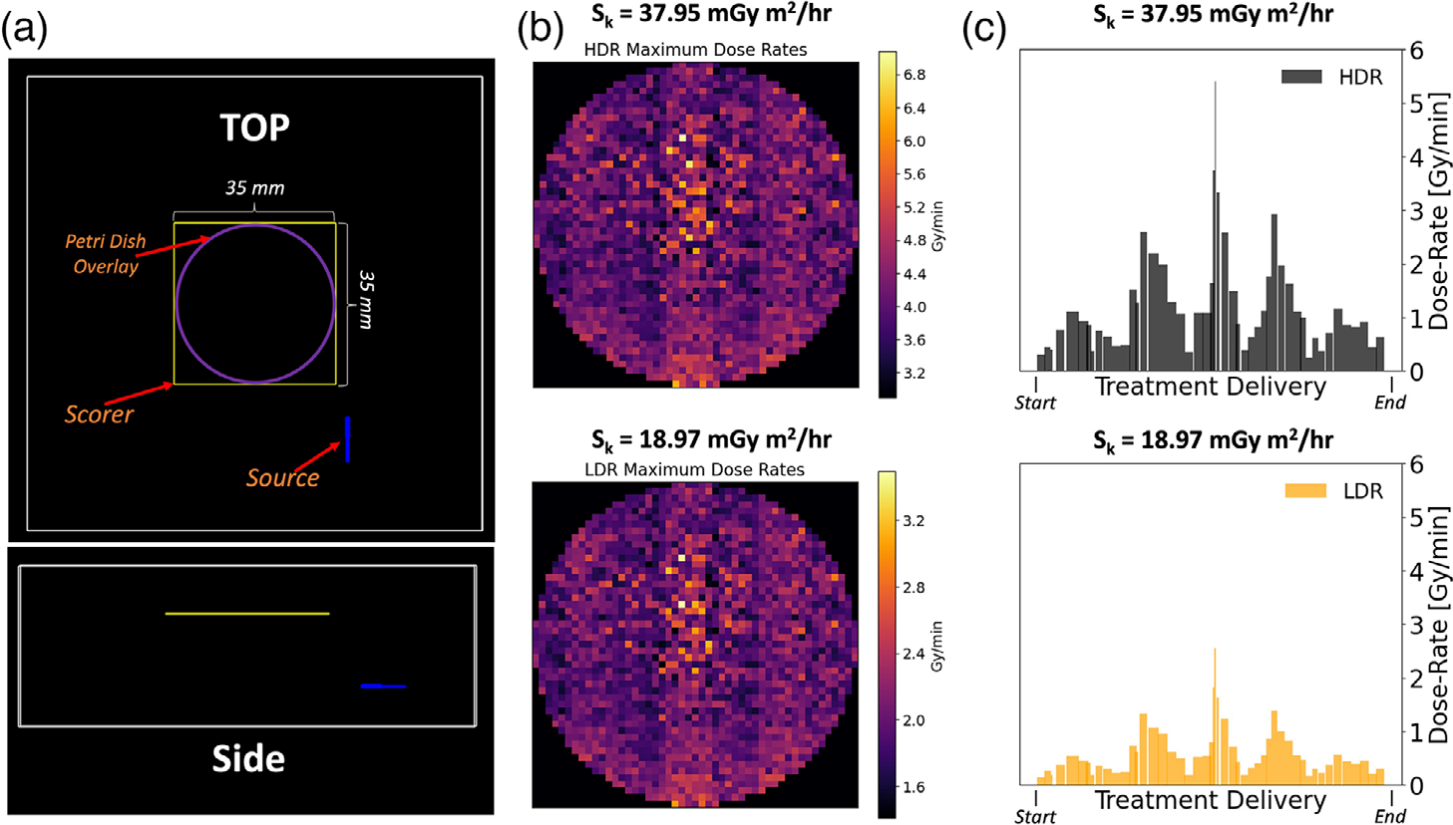
Temporal variability in dose rate delivery from 192‐Ir source. Monte Carlo simulation setup of Solid Water phantom with the scorer, petri dish, and source (a). Maximum dose rates across the face of the petri dish from an 192‐Ir source with high strength (37.95 mGy m^2^/h) and low strength (18.97 mGy m^2^/h) (b). Dose rates during treatment delivery at center of petri dish during both high and low‐source strength 192‐Ir irradiations (c).

To compare instantaneous dose rates between LINAC and HDR‐BT irradiations, the Solid Water phantom, along with the 192‐Ir Flexisource and the source strength, were simulated using MC platform TOPAS (Figure 6a).^29^ The maximum dose rates achieved across the face of the petri dish as measured using MC simulations varied between 1.4 to 3.5 (avg. 2.0) Gy/min for a source strength of 18.97 mGy m^2^/h, and between 2.8 to 7.0 (avg. 4.0) Gy/min for a source strength of 37.95 mGy m^2^/h (Figure 6b). As shown in Figure 6c, there are dose rate variations during treatment plan progression at the center of the petri dish, which clearly shows that the dose rate fluctuates significantly as the source proximity changes.

## 4 DISCUSSION

The purpose of this manuscript was to evaluate the dose rate dependence on GNP radiosensitization under variable dose rate delivery from HDR‐BT source decay. Furthermore, we matched the delivered dose rate from HDR‐BT during LINAC irradiations to compare the induced radiosensitization under variable incident photon energy. While other manuscripts have been published elsewhere regarding the dose rate dependence on GNP radiosensitization, no such study has thus far been conducted regarding the impact of 192‐Ir source decay on the measured radiosensitization with clinically plausible GNP concentrations. The impact of this study will be to inform future clinical treatments on the possible influence of the radioactive source strength on the outcomes of treatments employing GNPs as radiosensitizing agents. Treatment plan optimization may well be impacted in future clinical treatments. Due to the proximity of the source to the exposed tissue with or without internalized GNPs, voxelized dose rates or predicted SER could improve plan accuracy. However, before this occurs, the efficacy of the GNP platform should first be demonstrated in vivo and in preliminary clinical trials.

The measured clonogenic loss and DNA DSB induction follow similar trends, indicating minimal difference in LINAC and HDR‐BT irradiations on GNP‐treated samples. This result is counter‐intuitive as GNPs are hypothesized to be more beneficial in lower‐energy radiotherapy treatments, which indicates a biological rather than physical mechanism of radiosensitivity. These factors could be based on cell‐specific characteristics like DNA damage repair rate or concentration of ROS scavengers.^45^, ^46^, ^47^ Previous work investigating GNP‐radiosensitization between HDR‐BT and LINAC irradiations has shown that HDR‐BT induced significantly greater DNA DSBs and loss in cellular viability;^26^ however, the study investigated the effects on HeLa and PC3 cells and did not match dose rates between the two irradiation modalities. Therefore, when investigating the clinical translation of GNPs into HDR‐BT treatments, the efficacy of the GNPs for the particular treatment site with the available source strength should be investigated to determine their radiotherapeutic efficacy.

The investigation by Morozov *et al.* ^18^ found that while smaller ∼12 nm GNPs exhibited a dose rate response on their induced radiosensitivity, larger ∼20 nm GNPs exhibited an inverse relationship, whereby larger dose rates reduced the radiosensitivity. The authors do not offer an explanation for the competing results, though the results do demonstrate that for an individual NP platform, they should be carefully investigated for their implementation into a wide array of clinical scenarios.

While the instantaneous dose rates are significantly greater during LINAC irradiations, this does not necessarily translate to a proportional increase in radiosensitization, as we have reported. With lower‐energy irradiations, such as from HDR‐BT, a greater photoelectric cross‐section with GNPs could induce a greater concentration of photoelectrons, resulting in increased intracellular damage. This effect could be replicated with higher energy radiation delivery coupled with a significantly greater instantaneous dose rate. For instance, the maximum instantaneous dose rate from the LINAC irradiations were approximately 150–300 times greater than the maximum dose rates achieved with HDR‐BT irradiations. In contrast, the photoelectric absorption of GNPs from photons of energy ∼380 keV is approximately 175 times greater than that from 1.3 MeV photons, according to the NIST database,^48^ for equivalent GNP mass concentrations. These results could indicate that the average dose rate during treatment plan delivery plays a more significant role than the maximum instantaneous dose rate in induced GNP radiosensitization, an important finding for the incorporation of GNPs into treatment plans with significant dose rate variability. Previous discussion on the dose rate effect determined that the total treatment delivery time plays a greater role than both the average or instantaneous dose rate on tumor cell survival.^49^ However, our results presented here are the first to offer an argument on the relative importance on dose rate parameters when incorporating GNPs.

It should be emphasized that these experimental results do not explicitly evaluate whether the average or instantaneous dose rate has a greater effect on GNP radiosensitivity but rather lead to suggestions and recommendations for future research. The LINAC irradiations were conducted with a constant pulse rate of 100 Mu/min, though further experiments should determine if the pulse rate could simply be reduced to 50 MU/min to reduce the averaged delivered dose rate. These results will be investigated in future research evaluating the pulse‐rate dependency on GNP radiosensitization. Careful consideration should be made in these future experiments for the measurement of averaged versus instantaneous/maximum dose rate over time. For example, Figure 6 demonstrates that the maximum instantaneous dose rate received by the base of the petri dish fluctuates significantly across its face. If instantaneous delivered dose rate is found to significantly influence GNP‐induced radiosensitization, spatial variations across the petri dish and cell culture (as seen in Figure 6) must be carefully considered. Unlike LINAC irradiations, which deliver relatively uniform dose rates across the petri dish, different cells may experience substantially different maximum instantaneous dose rates during HDR‐BT delivery.

Other factors that could influence GNP‐induced radiosensitization include, but may not be limited to, mitochondrial damage, chemical enhancement, and other reactive oxygen species generation.^19^, ^50^ Previously, Roselli *et al.* ^51^ discussed a novel mechanism for GNP dose enhancement as a catalytic‐like reaction at the water‐nanoparticle interface based on the energy deposition in the surrounding medium, which they showed to contribute to hydroxyl radical production to a greater extent than energy deposition in the nanoparticle alone using similar GNP dosing concentrations. The authors further discuss hydroxyl radical production as a function of dose rate. They show that intra‐cellular media levels of the free‐radical appear to reduce with increasing dose rate, which they attribute to rapid recombination of the hydroxyl radicals at the surface of the nanoparticle, therefore reducing the amount of radicals re‐entering the surrounding media. This effect may be dependent on the surface charge of the nanoparticles. Therefore, the conjugation of neutral PEG to the surface of the nanoparticles, increasing the surface charge of the GNPs themselves, as performed in this study, may, in fact, promote hydroxyl radical formation in the intracellular medium. To deconvolve the influence of physical and biological factors on GNP‐induced radiosensitization, additional experiments should be conducted using specific biological probes. For example, ROS generation can be elucidated using coumarin based assays or mitochondrial superoxide indicators for both intracellular and extracellular ROS production.^20^, ^51^, ^52^

While the LQ model was employed in this work, other models may be more suited for modeling GNP‐radiosensitization. To this end, we chose to include the fit of a MTSH and TC model onto the HDR‐BT clonogenic survival for the purpose of this discussion. The MTSH model assumes the cell as *n* sub‐targets which should each be inactivated to cause cell death. The TC model attempts to improve on the MTSH at low doses by adding an additional single‐target component to the multi‐target model. Both models were fitted up to the 8 Gy irradiation data, given that these models could extend to greater doses compared to the LQ‐model. As shown in Figure S7a,b both the MTSH and TC model fit the HDR‐BT data well with R^2^ values between 0.97 and 0.99, similar to those observed for the LQ model. Other studies have also attempted to fit GNP radiosensitization to local‐effect models (LEMs) or a microdosimetric kinetic (MDK) model with success under certain conditions.^53^, ^54^ GNPs lead to highly heterogeneous dose spikes, which LEMs or MDKs attempt to model. However, many of these models do not consider radiobiological implications of GNPs. Furthermore, at the concentrations of GNPs employed in this research, no dose enhancement is expected based on Monte Carlo simulation results.^25^, ^55^, ^56^ Therefore, it is hypothesized that the radiosensitivity measured in this study is primarily due to biological influences that have yet to be fully modeled. These results suggest that a “one‐size‐fits‐all” approach to modeling GNP radiosensitization may not be necessary, but rather multiple models may prove beneficial in different situations. Further investigation into this area should be conducted.

## 5 CONCLUSIONS

GNP‐induced radiosensitization is a complex and multifaceted mechanism that is only now being further understood. The full landscape of their physicochemical properties and biological interactions must be well understood for their successful implementation into RT workflows. We have successfully shown the first in vitro dose rate effect from GNPs using clinically plausible nanoparticle concentrations and prescription doses for 192‐Ir and 6 MV LINAC irradiations with matched averaged dose rates. We showed that a higher strength 192‐Ir source coupled with GNPs can induce approximately 19% greater loss in cellular survival and 28% greater DNA damage compared to a low strength source. Similarly, for LINAC+GNP irradiations, a greater dose rate corresponded to a 13% greater SER than a lower dose rate and 22% greater DNA damage. Furthermore, we showed that the average dose rate during treatment delivery could be more influential in GNP‐induced radiosensitization than in the maximum instantaneous dose rate. Our results indicate that higher dose rates and increased photoelectric absorption at lower kilovoltage photon energies are ideal conditions to elicit significant GNP radiosensitization. While our results demonstrate minimal radiosensitization during HDR‐BT irradiations with a cold source, further work with more complex biological systems, such as spheroids or mice models and additional tumor cell phenotypes should be performed to characterize GNP‐induced radiosensitization during radioactive source decay further.

## CONFLICT OF INTEREST STATEMENT

The authors declare no conflicts of interest.

## Supporting information




**Supporting File 1**: mp70372‐sup‐0001‐SuppMat.pdf.

## Data Availability

The datasets used and/or analyzed during the current study are available from the corresponding author on reasonable request
