# Supplementary material for: Influence of radiotherapy dose rate on gold nanoparticle‐induced radiosensitization from high dose‐rate brachytherapy and external beam therapy
Source: Med Phys. 2026 Feb 27;53(3):e70372. doi: 10.1002/mp.70372 (PMC12949370; doi:10.1002/mp.70372)
Supplement: Supplementary file 1 — Supporting File 1: mp70372‐sup‐0001‐SuppMat.pdf. [file MP-53-0-s001.pdf]

Influence of radiotherapy dose rate on gold nanoparticle-induced radiosensitization from high dose-rate brachytherapy and external beam therapy

*Daniel Cecchi[1], Nolan Jackson[1], Sacha Freeman[1], Kieren O'Neil[1], Mehran Goharian[1,2], Wayne Beckham[1,2], and Devika B. Chithrani[1,2,3]**

1. Department of Physics and Astronomy, University of Victoria, Victoria, BC, V8P 5C2, Canada.
2. British Columbia Cancer, Victoria, BC, V8R 4Z3, Canada.
3. Centre for Advanced Materials and Related Technologies (CAMTEC), University of Victoria, Victoria, BC, V8P 5C2, Canada.

*Corresponding Author:

Email: devikac@uvic.ca

Mailing Address: 3800 Finnerty Rd, Victoria, BC, V8P 5C2, Elliot Building

Supplemental Figure 1:

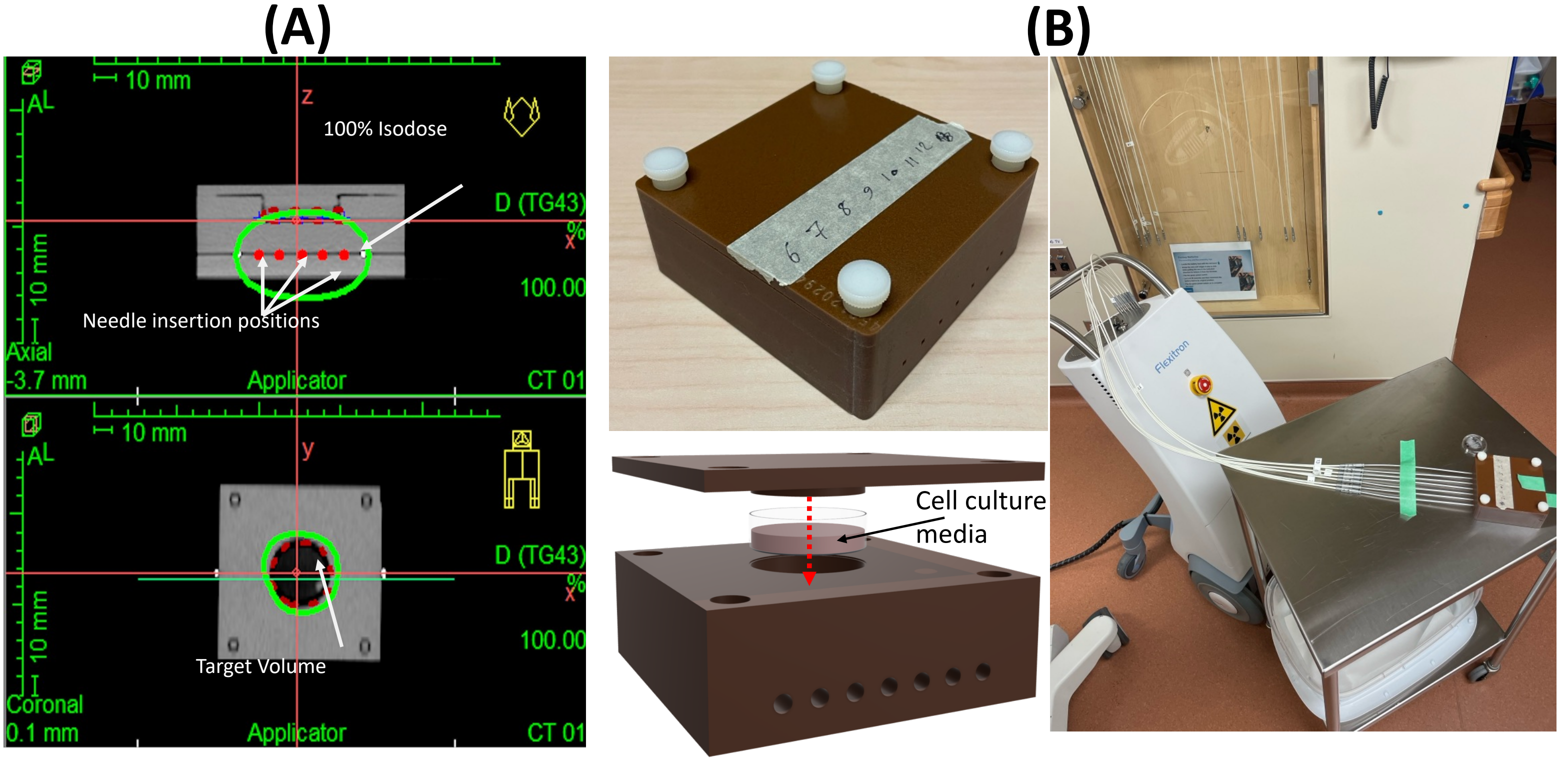

Figure S1: Treatment planning and radiochromic film experimental setup. (A) CT-scan of phantom in Oncentra treatment planning system with 100% isodose line depicted in green, and the target volume as the red-dashed line. (B) Solid Water phantom and HDR treatment setup.

Supplementary Figure 2:

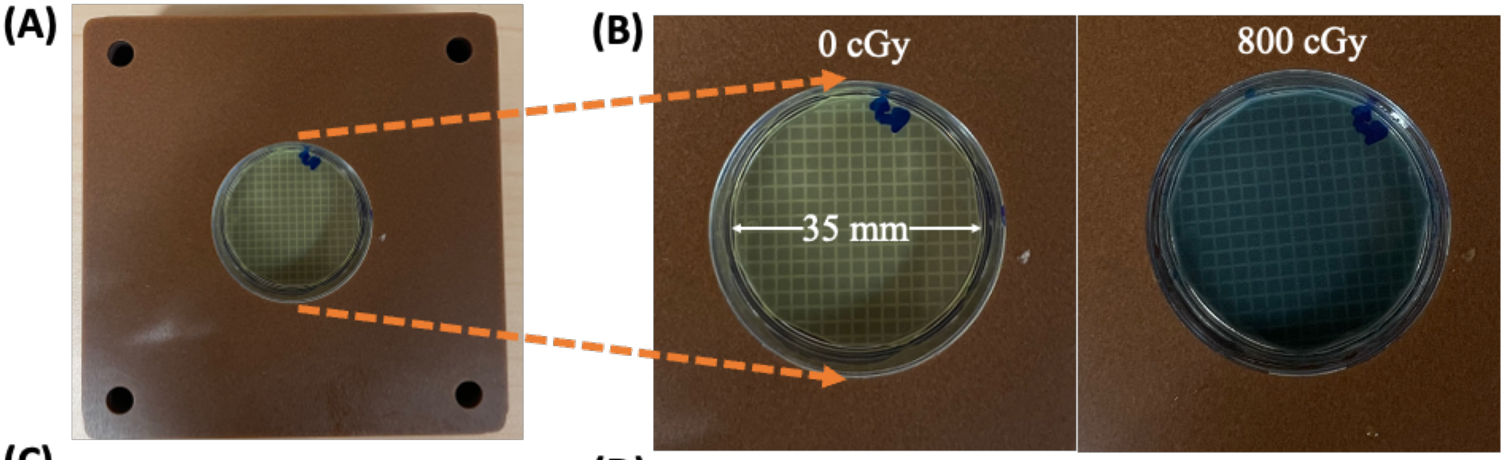

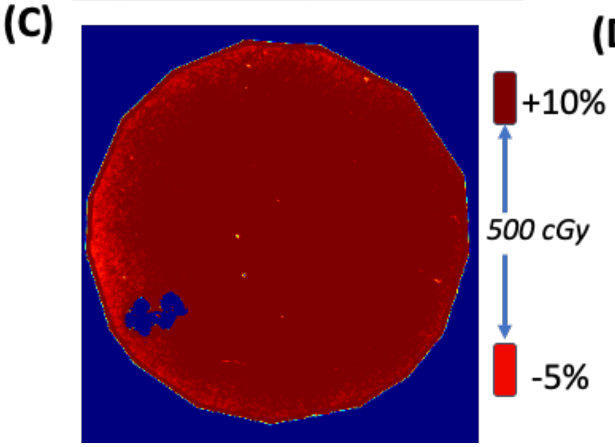

(D)

TG-43 [cGy]	Film [cGy]	Flatness [%]		Symmetry [%]	
		H	V	H	V
500	503.2	2.1	3.6	-0.6	1.5
800	808.5	8.3	4.9	-2.4	0.5

Figure S2: Radiochromic film radiation treatment and dosimetry. (A) Cut EBT-4 radiochromic film within phantom. (B) Radiochromic film before (left) and after (right) 192-Ir 800 cGy radiation delivery. (C) Scanned 500 cGy irradiation film. (D) Mean dose delivery and flatness and symmetry of horizontal (H) and vertical (V) film profiles.

<u>Quantification of Gold Nanoparticle Content in Intracellular Matrix</u>

Absolute gold content per cell was calculated using the following equation:

$$\frac{Gold\ Nanoparticles}{Cell} = \frac{\left(Gold\ Concentration\ Per\ Sample\ \left[\frac{g}{mL}\right]\right) \cdot (Sample\ Volume\ [mL]) \cdot \left(N_A \left[\frac{atoms}{mol}\right]\right)}{\left(Gold\ Atomic\ Mass\ \left[\frac{g}{mol}\right]\right) \cdot (Number\ of\ Cells) \cdot \frac{Gold\ Atoms}{Gold\ Nanoparticle}}$$

The gold concentration per sample, sample volume, and number of cells per sample vary between conditions and individual samples; the atomic mass of gold is 196.96657 g/mol; N_A is Avogadro's number – $6.022\text{x}10^{23}$ atoms/mol; the number of gold atoms per nanoparticle varies depending on the size and is calculated based on the following equation:

$$\frac{Gold\ Atoms}{Gold\ Nanoparticle} = \frac{(Atoms\ per\ unit\ cell) \cdot (Gold\ Nanoparticle\ Volume\ \ [nm^3])}{Unit\ Cell\ Volume\ [nm^3]}$$

$$= 4 \cdot \frac{\frac{4\pi r^3}{3}}{a^3} = \frac{2}{3}\pi\left(\frac{D}{a}\right)^3$$

Where D = 11 nm is the core diameter of the gold nanoparticle, and a = 0.408 nm is the length of a unit cell of gold atoms.

Supplementary Figure 3:

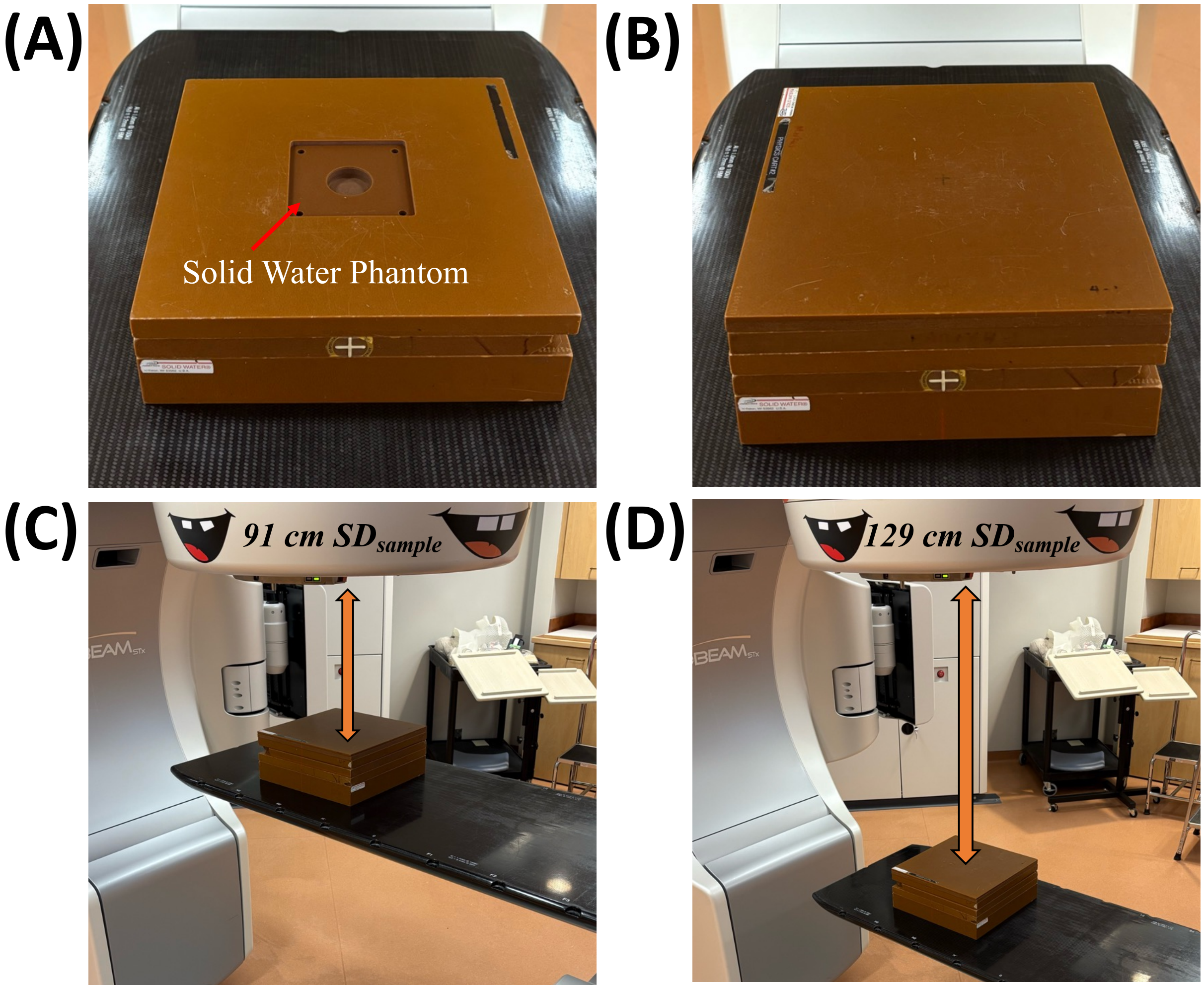

Figure S3: Treatment delivery setup on clinical LINAC. Solid Water Phantom with expansion to incorporate greater photon scatter from high-energy radiation (A-B); Calculated source-sample distances (SD_{sample}) for high dose rate (C) and low dose rate (D) treatment delivery.

TMR-Based Monitor Unit Hand Calculations to Determine Required Source-to-Axis Distance for LINAC Irradiations

TMR-based monitor unit (MU) hand calculations were performed using the following formula:

$$MU = \frac{Dose\ [cGy]}{\frac{1\ cGy}{MU} \cdot TMR(w_m, d) \cdot S_C(w_c) \cdot S_p(w_m) \cdot \left(\frac{Source - Sample - Distance}{SAD}\right)^2}$$

TMR is the tissue maximum ratio at the specified depth (d) and field size at measurement (w_m), S_c is the collimator scatter factor given the collimator field size (w_m), S_p is the phantom scatter factor at a given field size at measurement (w_m), and the last factor is an inverse square correction.

These factors are listed for each treatment unit at the clinic for a given depth or field size. To determine the required source-sample-distance (SD_{sample}) to achieve either 110 or 55 cGy/min, the distance was iterated between 90 cm to 200 cm in 220 increments, correcting the field size (w_m) for each location using the inverse square law and performing linear interpolation where required between successive data points. The calculations were then reversed to determine the required MU to deliver either 200, 500, or 800 cGy.

	Dose Rate [cGy/min]	Sample - Distance [cm]	MU		
			200 cGy	*500 cGy*	*800 cGy*
110 cGy/min	109.7	91.6	182.3	455.8	729.2
55 cGy/min	55.1	131.6	362.9	907.5	1452

Table S1: Calculated dose rates from MU hand calculations and the corresponding source-sample distances along with total delivered MUs for 200, 500, and 800 cGy.

A cylindrical PTW ion chamber (SN: 2217) and electrometer were used to validate the calculated source-sample distance. The ion chamber was placed at the reference 5 cm depth and 100 cm SAD to replicate the sample location and irradiated to 220 MU as calculated by Eclipse to deliver a 200 cGy dose to the petri dish at isocentre. After this, the total collected charge was recorded in triplicate and averaged. The treatment setup was then raised or lowered to the predicted SD_{sample} of 91.6 and 131.6 cm and irradiated to 362.9 or 182.3 MU, respectively. The total collected charge was recorded, and the SD_{sample} was then altered based on the recorded charge until the total MUs delivered the same total charge to the ion chamber at the reference location.

**Avg. Charge Reading [nC]**	Isocentre	Correct SD_{sample}	
		93.2 cm	133.7 cm
	37.26	37.35	37.25
% Diff Ref. Pt.	---	0.24%	-0.03%

Table S2: Total collected charge readings at the reference location and at modified SADs.

Supplementary Figure 4:

(A)

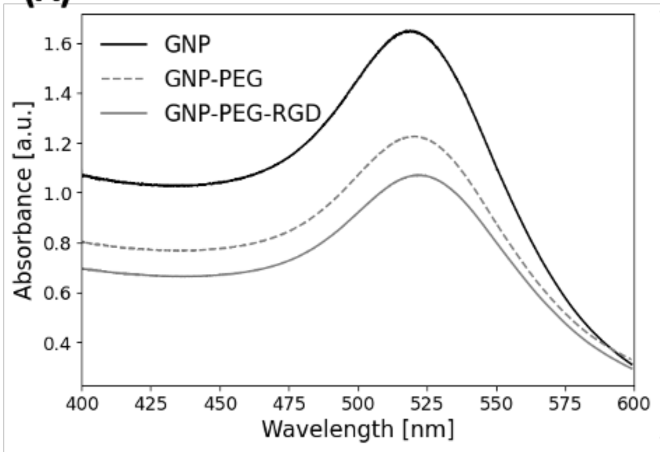

(B)

Sample	Diameter [nm]	Concentration [ug/mL]
GNP	11.7 ± 0.043	243.2 ± 61.1
GNP-PEG	12.5 ± 0.051	166.5 ± 35.3
GNP-PEG-RGD	13.7 ± 0.049	159.9 ± 27.1

Figure S4: Absorbance of colloidal GNP solution as a function of incident wavelength obtained from ultraviolet visible spectrometry at each step of the functionalization process. The diameter and concentration are interpolated from tabulated data.

Supplemental Figure 5:

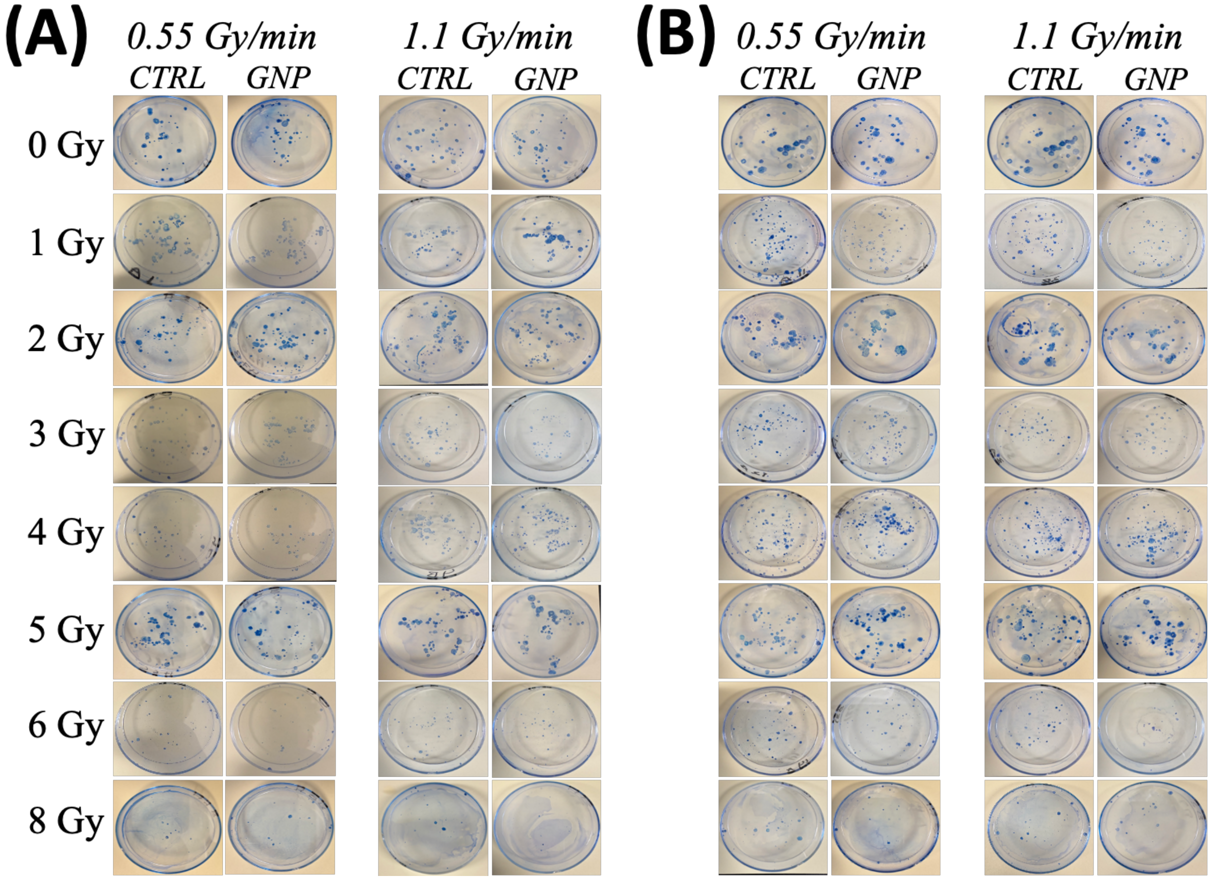

Figure S5: Colony formation of HEC-1A cells after irradiation from either HDR-BT (A) or LINAC (B) in high and low dose rate environments.

Supplemental Figure 6:

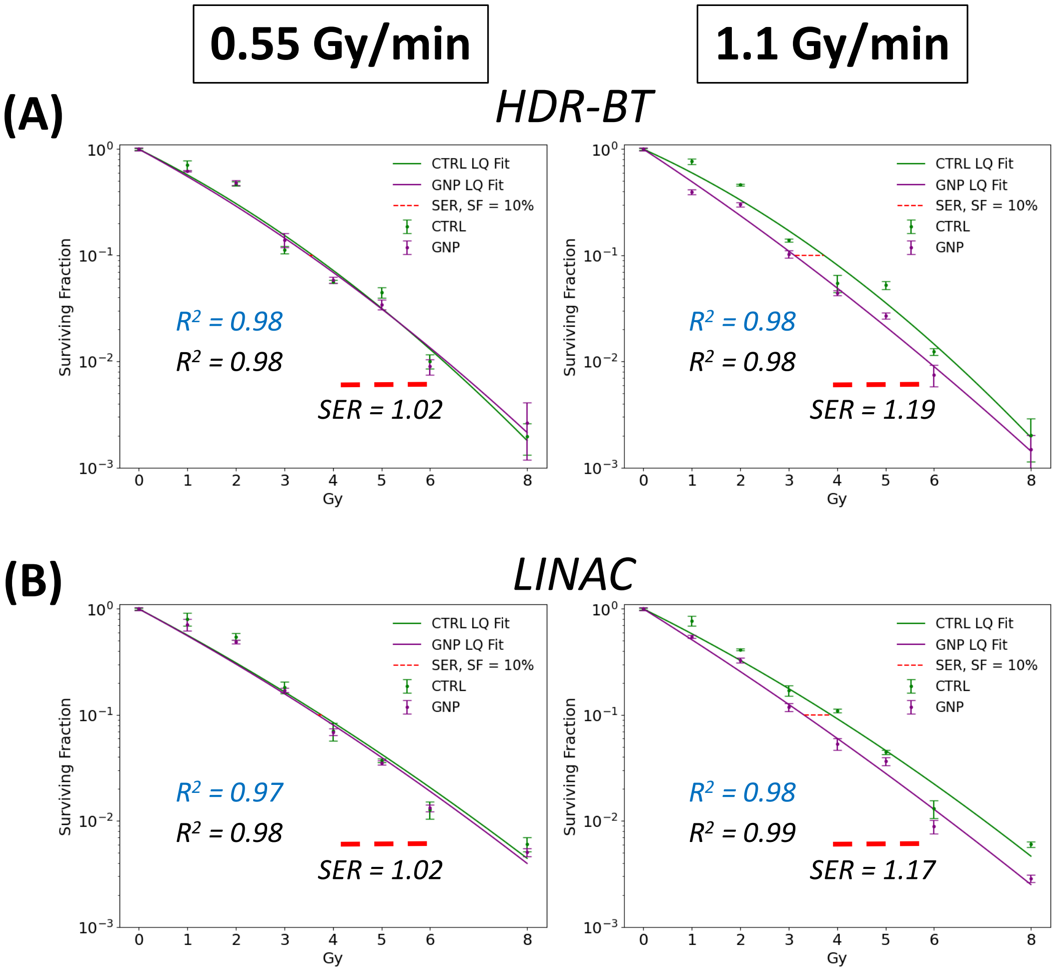

(A)

	CTRL	*GNP*
	1.1 Gy/min	
α	0.47	0.69
β	0.04	0.02
	0.55 Gy/min	
α	0.52	0.57
β	0.03	0.03

(B)

	CTRL	*GNP*
	1.1 Gy/min	
α	0.52	0.66
β	0.02	0.01
	0.55 Gy/min	
α	0.55	0.56
β	0.02	0.02

Figure S6: Cell survival curves of GNP-treated and untreated cells post HDR-BT (A) and LINAC (B) irradiations at 0.55 Gy/min and 1.1 Gy/min for treatment doses between 0 – 8 Gy. (B)

Supplementary Figure 7:

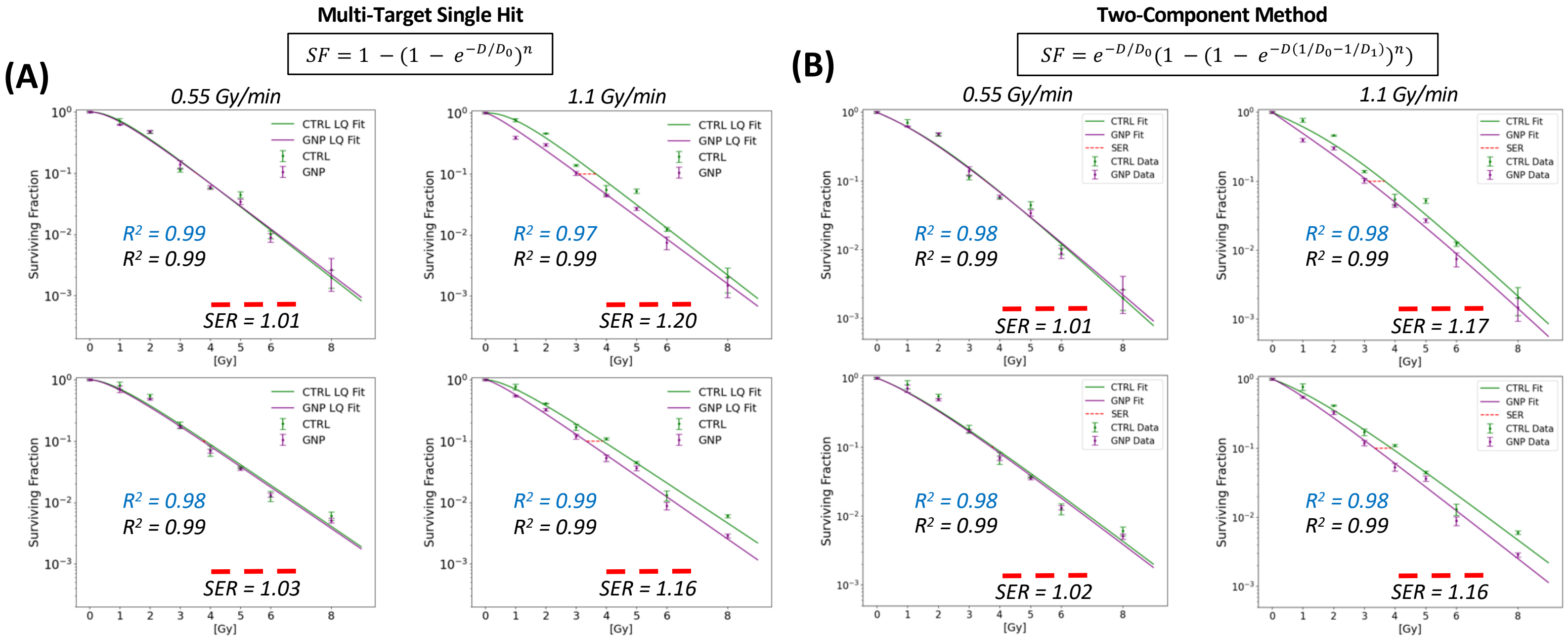

Figure S7: (A) Multi-target single hit model fit and (B) Two-component method fit to HDR-BT (Top) and LINAC (Bottom) irradiations at 0.55 and 1.1 Gy/min between doses of 0 – 8 Gy.

Supplementary Figure 8:

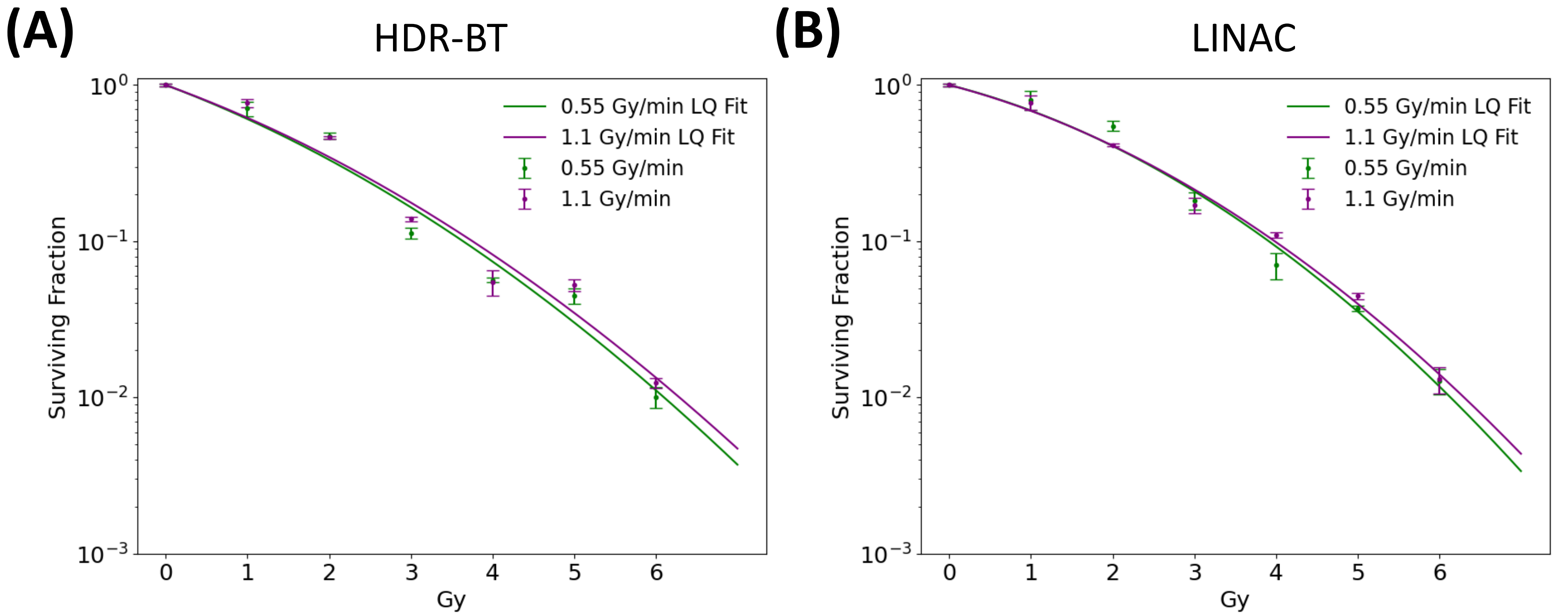

Figure S8: Cell survival curves without GNPs at 0.55 and 1.1 Gy/min for HDR-BT (A) and LINAC (B) irradiations.

Supplementary Figure 9:

(A) (B)

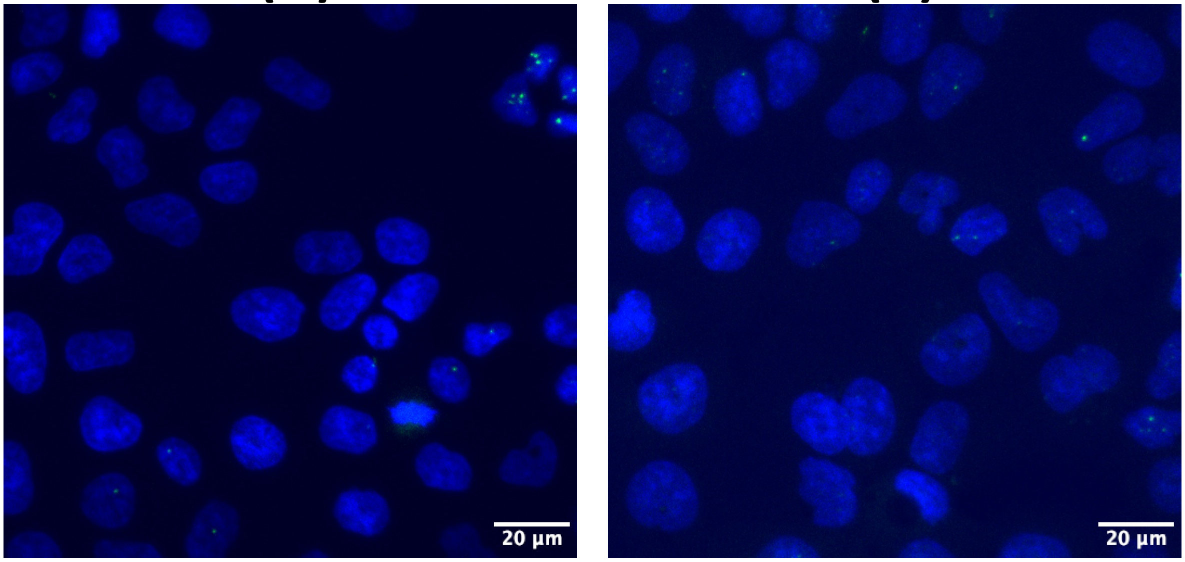

(C)

Counts per Cell	
Control	GNPs
0.21	0.3
$p > 0.05$	

Figure S9: Confocal images of unirradiated cells; nuclei are stained in blue with DNA damage sites in green. Control samples (A) and GNP-treated samples (B). Average foci counts per cell are statistically insignificant between conditions (C).
